# Method of Using Soft Foil

**Published:** 1898-05

**Authors:** Benjamin Lord

**Affiliations:** New York


THE
### International Dental Journal.

Vol. XIX. May, 1898. No. 5.

### Original Communications.[1]

### METHOD OF USING SOFT FOIL.[2]

BY BENJAMIN LORD, D.D.S., NEW YORK.

I AM asked to explain and demonstrate my method of using soft foil, which I ought to be quite willing to do, if for no other reason than that the Executive Committee has requested it.

It may be said that such demonstrations are a new departure in the proceedings of our meetings, except in a recent instance, but the subject is a thoroughly practical one, and I hope that many of our members and guests will be asked in due time to explain their methods. That will enable us to learn a great deal from one another of different styles and modes of operating, and by careful comparison and an earnest desire for that which is most effective in securing the best results, much progress may be secured.

Notwithstanding the many meetings dentists hold, and the social and friendly intercourse which they highly enjoy, they really know very little about one another's methods of filling teeth, or the instruments they use for the purpose. It is strange that there is no

---

[1] The editor and publishers are not responsible for the views of authors of papers published in this department, nor for any claim to novelty, or otherwise, that may be made by them. No papers will be received for this department that have appeared in any other journal published in the country.

[2] Read before The New York Institute of Stomatology, Tuesday evening, March 1, 1898.

standard among us, but every dentist has his own peculiar way of working, and it has been said that very few change much from seeing or from being told of other and perhaps better ways and methods, but I do not believe that the saying is true.

There must be a best way of doing almost everything, notwithstanding that so much precision and accuracy are required in the work of filling teeth, yet we find that good and successful operations are made by different operators, who use quite different methods and instruments.

This, of course, does not prove that all the methods practised are equally good, but it does prove that the individual methods may for the time being at least be the best for those using them.

I propose to have a sort of familiar talk with those present, and it will be understood that it is not my purpose to show how to fill teeth, but to tell how I do it. I will speak of the preparation of cavities, of the instruments I use for the purpose, and how I use them; but will not, for the want of the necessary facilities, attempt to prepare cavities. This is beyond what I was requested to do, but I have felt that it might not be without interest, as the success of a filling depends so much upon the proper preparation of the cavity.

For instruments I depend largely upon what is known as the hatchet excavator. The blades, of course, must be of different lengths and widths and curved at different angles, some having also a slight bend in the shank. Some are wider at the cutting edge than at the angle of the curve, others have the sides or edges parallel, and some of both these classes should have one or both corners ground off so that the cutting edge would be rounded. Then I use a cutting instrument of a peculiar shape, which is most effective on the labial and buccal surfaces, and on the approximate surfaces where the spaces are considerable.

This instrument is specially adapted for preparing and shaping the walls or sides of the cavity, and is not intended to be used on the floor of the cavity.

I use burs very little except to open crown cavities and dress margins, the proper preparation of which I consider of the greatest importance to the accuracy and success of the fillings.

I have, as can be seen, instruments cut much as burs are, for dressing away the edges of the cavity, after the removal of the decay, and for giving the cavity a proper shape, including a slight undercut.

These instruments, if properly used, take away all decomposed structure and very thin edges, leaving margins of a thickness not likely to be crushed or powdered in any part of the operation of filling. Then I use a suitable instrument to give the edge of the cervical wall a slight bevel outward, so as to bring the filling slightly over the margin of the cavity. I would also thus bevel other margins that are thin.

I feel that the dental engine is used very often to the destruction of healthy tooth-structure and the marring and notching of the margins of cavities. It is unquestionably a very useful appliance, and it is also a very harmful one when used improperly or where it ought not to be.

I prefer soft foil because of its easy adaptation to the walls of the cavity and the greater ease and certainty with which it can be placed and packed without danger of tipping from becoming hard too soon, which is often true of cohesive gold.

Cohesive foils and other preparations of cohesive gold have their uses, such as the restoration of broken corners or the sides of the teeth, where it is desirable to make such restorations, but I may say I think it is done quite too frequently, particularly with the front teeth, thereby giving them a more unsightly appearance than the marred or broken corners would present.

My experience and observation lead me to believe that cohesive gold is not so well adapted for general use as soft gold.

In my judgment the best form in which to use gold is in sheets, parts of the sheet being folded in strips of suitable widths for the cavity in which they are to be used. I consider that strips or folds can be handled with more ease and certainty in the process of filling than can rolls, pellets, or blocks.

It is my belief that too many forms of gold are prepared for our use, often leading to confusion and embarrassment, particularly on the part of the inexperienced. If my memory serves me correctly, the late Mr. R. S. Williams advertised that he prepared and had for sale one hundred and fifty different varieties of gold for dentists' use.

Filling teeth has so many difficulties to be overcome, and so much accuracy and fidelity are required, that we must follow certain lines for a long while, and, in the use of particular forms of gold, to arrive at a good degree of excellence.

By using suitable points all cavities having three walls can be filled and the fillings contoured by the use of soft foil; this may be done with equal certainty and success with tin-foil or with a combination of gold and tin.

I depend mostly upon a single form of instrument for placing the gold and for condensing it towards the walls of the cavity. Occasionally I use a point somewhat more curved to carry the gold already placed, with more force against the wall or into a corner of the cavity (always guarding against too much pressure), so that each additional layer may not only be placed against that which is already in position, but will be carried into it, thereby uniting the whole mass.

Then I come to the surface condensing, which I begin with a small four-sided instrument, curved to an angle of forty-five degrees and pointed, the sides grooved, and the edges of the grooves serrated.

I assume, as must be the case to insure the best results, that the foil has been accurately placed, the layers added in regular order, and condensed as solidly as possible by lateral condensing, and that the gold is raised sufficiently above the margins of the cavity, so that after the surface is made solid by using the point of the instrument and the edges of the groove, the filling will still have a contour or oval surface, if an approximate filling. I wish to attach great value to this surface condensation with small points, as it more or less spreads the filling to some extent towards the walls of the cavity, and ties the whole mass together more securely. I speak of leaving the surface of the filling contoured or oval, which is certainly most natural, and, if so, highly desirable, but there is interest in the old style of the flat surface, or even in the concave surface, as there is much more certainty that the gold will be made solid against the walls of the cavity, with less care, when the surface is thus worked down. We find that fillings so finished have done most excellent service.

But by using points and making the pressure near the margin of the filling, so as to condense against the walls, and also using the edge of the groove of the instrument I have alluded to, full success may be secured, when the surface is left contoured.

I now come to the use of the broad instrument with one side flat and the other somewhat oval, both for further condensing and burnishing. The oval side will not be so likely to come in contact with the margins, and can be used to greater advantage to condense the surface; the flat side also possesses advantages over the oval for a part of the work.

I am now ready to give the margins their finish, and I think it will be seen that my instruments are well adapted for the purpose. They have one side flat and the other oval, are cut like burs, and are used much as a file of the same shape would be. For the final finishing I use a small instrument, the same as I use for the removal of tartar or any roughness about the teeth, and if there should still be any surplus gold at the margins, this instrument will be sure to detect and remove it.

I attach little if any value to the mere burnishing of the surface of fillings, only so far as it gives greater solidity to the surface. The great point in the finishing of fillings is the smoothness and solidity of the margins, so that no surplus material be left overhanging.

I do not think that any kind of a disk, sand-paper, or corundum should be used on the approximate surfaces for finishing fillings, as they are liable to cut away tooth-material or filling that ought not to be removed, and I do not like the use of the polishing strips, for the same reason. I also think that a bur or burnisher in the engine should never be used to reduce or polish fillings in the grinding surfaces of the teeth, as there is too much danger of reducing the cusps or injuring the margins of the cavity.

The grinding surfaces should be finished to correspond in irregularity to the natural surface of the tooth, and instruments may be used for that purpose. The burnishing is really of no importance except to give solidity.

It will be observed that I have gone into quite a good deal of detail, perhaps more than was required or in good taste, except, perhaps, to students, but it seemed to be necessary in order to cover the ground. Of course, a great deal has been left unsaid, and it is earnestly hoped that the question may be considered only just opened, and the subject in all its aspects and bearings may be thoroughly discussed, so that we may if possible determine some of the causes of so much failure and correct them.

I will now show my instrument and my manner of preparing and handling the gold.

I use Abbey's foil, and prefer No. 5. I cut a sheet generally into four pieces; if the cavity is small, I would cut the sheet into six pieces. I fold each piece into strips, for the larger cavities about an eighth of an inch wide, and for the smaller ones a sixteenth of an inch.

For the folding I use one side of my shears, instead of a table-knife or a paper-cutter, and I fold on a small cushion made of soft leather and filled with wool. I prefer this to anything harder. The strips are placed on a folded napkin, and I use the point of the instrument with which I am working to pick up the strip and carry it to the cavity. I usually cut the strips into two pieces, sometimes three. The instrument that I use and depend upon mostly for placing, uniting, and condensing the folds is properly curved at the end, and an absolute point made by a very short bevel on three sides with a sharp file, and the fourth side touched at the point very slightly with the file, so that the line of the curve may not be changed.

I do not, as would be understood, condense with the point of the instrument, but with the side and edge of the bevel, and unite the folds with the point.

I strive as far as possible, in placing the foil in the cavity, to fold the strip in so as to form a loop and bring the loop to the surface.

I prepare and use tin-foil the same as I do gold, and also gold and tin in combination. I use three parts gold and one of tin when combining the two metals, folding them together with the gold outside.

I hold in my hand a tooth having a cavity with four walls, which, of course, is the easiest kind to fill. I begin by putting the first piece against the most distant part of the cavity, and the second piece against and into the first piece with a hard, firm pressure, and so on against the walls, and filling in the centre as is required, to the end.

To fill a compound cavity or one with three walls only, I would pursue the same method, by placing, uniting, and packing against the walls and filling in the centre, and finish by building up where the wall of the cavity was wanting.

